# α7 Nicotinic Acetylcholine Receptor May Be a Pharmacological Target for Perioperative Neurocognitive Disorders

**DOI:** 10.3389/fphar.2022.907713

**Published:** 2022-06-03

**Authors:** Penghui Wei, Wenyuan Lyu, Lin Xu, Hao Feng, Haipeng Zhou, Jianjun Li

**Affiliations:** ^1^ Department of Anesthesiology, Qilu Hospital (Qingdao), Cheeloo College of Medicine, Shandong University, Qingdao, China; ^2^ Department of Anesthesiology, Qilu Hospital of Shandong University, Jinan, China

**Keywords:** neuroinflammation, α7 nicotinic acetylcholine receptor, brain-derived neurotrophic factor, aging, perioperative neurocognitive disorders

## Abstract

**Background:** The α7 nicotinic acetylcholine receptor (α7nAChR) is a promising therapeutic target in neurodegenerative diseases. This study examined the effects of surgery and anesthesia on α7nAChR expression in the central nervous system and determined the mechanisms by which α7nAChR mediates neuroprotection in perioperative neurocognitive disorders (PNDs) in aged mice.

**Methods:** Eighteen-month-old male C57BL/6J mice underwent aseptic laparotomy under isoflurane anesthesia, maintaining spontaneous ventilation to establish the PNDs model. Agonists and antagonists of the α7nAChR and tropomyosin receptor kinase B (TrkB) receptors were administered before anesthesia. The α7nAChR expression, peripheral as well as hippocampal interleukin-1β (IL-1β), and the brain-derived neurotrophic factor (BDNF) levels were assessed. Separate cohorts of aged mice were tested for cognitive decline using the Morris water maze (MWM).

**Results:** Surgery and anesthesia significantly suppressed α7nAChR expression in the hippocampus and cortex. Surgery-induced IL-1β upregulation in the serum as well as hippocampus and hippocampal microglial activation were reversed by the α7nAChR agonist**.** A significant reduction in the hippocampal BDNF levels were also observed. The α7nAChR stimulation reversed, and α7nAChR suppression promoted BDNF reduction in the hippocampus. Blocking the BDNF/TrkB signaling pathway abolished α7nAChR-induced neuroprotection in PNDs, as evidenced by poor cognitive performance in the MWM test.

**Conclusions:** These data reveal that α7nAChR plays a key role in PNDs. The mechanisms of the anti-inflammatory pathway and BDNF/TrkB signaling pathways are involved in α7nAChR-meidiated neuroprotection in PNDs.

## Introduction

Aging increases the brain susceptibility to the stresses of surgery and anesthesia and impedes cognitive recovery, promoting neurocognitive disorders causing a major concern for these older individuals during postoperative procedures (Vacas et al., 2021). Perioperative neurocognitive disorders (PNDs), including acute postoperative delirium and long-term postoperative cognitive dysfunction, affect functional independence as well as increases mortality (Steinmetz et al., 2009; Leslie, 2017). Evidence suggests that PNDs have a synergistic effect on the neurodegenerative diseases and may accelerate pre-symptomatic Alzheimer disease (AD) as well as related dementia (Yang et al., 2020). Importantly, severe acute respiratory syndrome coronavirus 2 infection may potentially worsen cognitive decline associated with anesthesia as well as surgical stress through synergistic mechanisms, and PNDs may present a notable and continued increase during the coronavirus disease 2019 pandemic (Wei et al., 2021; Jain et al., 2022).

The pathophysiology of PNDs and potential targets for their prevention and treatments remain to be explored. Perioperative neuroinflammation plays a critical role in development of PNDs and anti-inflammatory interventions during or after surgery may be very attractive. However, the immune response induced by surgical and anesthetic stress is triggered to protect the brain and restore homeostasis, and anti-inflammatories may disrupt the positive effects of neuroinflammation leading to immunosuppression and delayed healing (Yang et al., 2020). Therefore, therapeutic interventions that boost the body’s anti-inflammatory pathway that includes the vagus nerve, to break the immune system overactivation and inhibit dysfunctional neuroinflammation may offer a reliable and safer approach to prevent and treat PNDs (Terrando et al., 2011; Leslie, 2017).

The cholinergic anti-inflammatory pathway can directly modulate the inflammatory response, which is reportedly important in resolving the inflammatory pathogenesis of several diseases, including sepsis, rheumatoid arthritis, and inflammatory bowel disease (Borovikova et al., 2000; Terrando et al., 2011). Furthermore, preoperative anticholinergics (scopolamine) and medications with anticholinergic properties (benzodiazepines) have clinically deleterious effects on neurocognitive function and increase the risk of developing PNDs (Mahanna-Gabrielli et al., 2019; Vacas et al., 2021). Nicotinic acetylcholine receptors (nAChRs) play a central role in the cholinergic pathways. Our previous studies have indicated that nicotine, a non-selective nAChRs agonist, activates the brain-derived neurotrophic factor (BDNF)/tropomyosin receptor kinase B (TrkB) signaling pathway, inhibits the NF-κB signaling pathway in the hippocampus, and reverses PNDs in aged rats (Wei et al., 2018). However, the role of α7 nAChR, one of the most commonly targeted receptor subtypes in neurodegenerative diseases (Hoskin et al., 2019), and its potential mechanisms underlying remain unknown. In this study, we investigated effects of surgery and anesthesia on α7nAChR expression in the central nervous system (CNS) and determined the mechanisms by which α7nAChR mediates neuroprotection in PNDs in aged mice.

## Materials and Methods

### Chemicals

PHA 568487 (PHA, an α7nAChR agonist) was purchased from Santa Cruz Biotechnology (sc-204186; Dallas, TX, United States). Methyllycaconitine (MLA, an α7nAChR antagonist) was purchased from Abcam (ab146684; Cambridge, United Kingdom). PHA and MLA were dissolved in 0.9% sterile saline and administered intraperitoneally (i.p.). K-252a (an antagonist of the TrkB receptor) was purchased from Sigma-Aldrich (K2015; St. Louis, MO, United States). The control group received the same volume of saline.

### Mice

All animal experiments were approved by the Animal Care and Use Committee of Qilu Hospital (Qingdao), Shandong University (approval NO: KYDWLL-202101) and performed in accordance with the guidelines for experiment animals use established by the Institutional Animal Care and Use Committee of Shandong University. Eighteen-month-old male C57BL/6J mice (Nanjing Junke Bioengineering Corporation, Ltd., Nanjing City, Jiangsu Province, China) were utilized. They were fed standard rodent food and water *ad libitum* and housed (five mice per cage) in an air-conditioned environment with 12:12 h light:dark cycles. All mice were acclimated to the environment for 7 days before the procedures.

### Establishment of PNDs Model and Drug Administration

**FIGURE 1:**
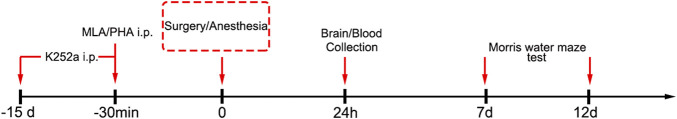
Experimental protocol. Aged mice were pretreated intraperitoneally with an antagonist of TrkB receptor (K-252a) 15 days before anesthesia. 30 min prior to anesthesia, the mice were administered intraperitoneally with an α7nAChR agonist (PHA) and antagonist (MLA). Blood and brain tissue were collected for ELISA, Western blot, and immunofluorescence detection, 24 h later. Another cohort of aged mice were randomly allocated to the four groups for MWM test from days 7 to 12 after surgery.

A modified aseptic laparotomy was performed under isoflurane anesthesia, while maintaining spontaneous ventilation, as described previously (Barrientos et al., 2012; Qiu et al., 2020). Anesthesia was induced in a chamber prefilled with 3.5% isoflurane and 100% oxygen. After losing righting reflex, mice were made to lie on the right side and anesthetized with 1.5% isoflurane, for 20 min. The surgery was performed under 1.5–2% isoflurane anesthesia and a facemask supplied 100% oxygen during surgery. A 1-cm midline vertical incision was made in the abdomen and the peritoneal cavity was penetrated and explored. The operator manipulated the viscera as well as musculature of the viscera and then exteriorized approximately 2 cm of the intestine and vigorously rubbed it using two sterile swabs for 30 s. The intestines were placed back into the peritoneal cavity and the wound was closed and sutured using 4-0 sterile surgical sutures. Polysporin (Johnson & Johnson Inc.) was used to relieve postoperative pain. The surgical procedure lasted for 15 min, and the total duration of isoflurane anesthesia was 1 h. Low-dosage of K-252a was selected to avoid side-effects and intraperitoneally administered 15 days before anesthesia (25 μg/kg per injection). Either MLA (4 mg/kg, i.p.) or PHA (low-dose 0.3 mg/kg or high-dose 0.6 mg/kg, i.p.) was administered 30 min before anesthesia. The dosages and timelines used were based on previous reports and our pilot study (Terrando et al., 2011; Hashikawa et al., 2017). A schematic illustration of the experimental procedure is outlined in Figure 1.

### Tissue Harvesting and Sample Preparation

Blood was collected *via* cardiac puncture under terminal isoflurane anesthesia in heparin-coated syringes and clots for 2 h at room temperature. The serum fraction was collected by centrifugation for 15 min at 2,000 g and 4°C. Immediately after blood collection, the whole brain was removed from the skull and hippocampal tissues were harvested on ice. Serum and hippocampi were stored at −80°C for enzyme-linked immunosorbent assay (ELISA) and/or Western blot analysis. The remaining mice were perfused with saline followed by ice-cold 4% paraformaldehyde into the left ventricle after transcardiac blood collection. The brains were removed and post-fixed in 4% paraformaldehyde overnight at 4°C, followed by rehydration, embedding in paraffin, and cutting coronally for immunofluorescence staining.

### Immunofluorescence

The sections were deparaffinized and rehydrated, followed by antigen retrieval by microwaving the sections in a sodium citrate buffer. Then, these sections were incubated in phosphate-buffered saline containing 3% hydrogen peroxide, to inactivate endogenous peroxidase, blocked with 3% bovine serum albumin at room temperature, and incubated overnight at a 4°C with rat monoclonal antibody α7nAChR (dilution 1:200, sc-58607; Santa Cruz Biotechnology, Dallas, TX, United States) and rabbit monoclonal antibody ionized calcium binding adaptor molecule 1 (IBA-1) (dilution 1:100, 10904-1-AP, Proteintech, China). After washing with phosphate-buffered saline three times, the secondary antibodies, goat anti-rat or goat anti-rabbit IgG-Cy3, were added for incubation. Subsequently, the sections were washed three times and incubated with 4′,6-diamidino-2-pheny-lindole (DAPI) for nuclear staining. Images were captured using a fluorescence microscope (Olympus, Japan) and assessed using ImageJ software (National Institute of Health, United States).

### Cytokine Measurements

The hippocampal tissue was homogenized on ice using a cell lysis buffer containing protease as well as phosphatase inhibitors and phenylmethanesulfonyl fluoride. Stored serum sample and hippocampal homogenate interleukin 1β (IL-1β) levels were measured using commercially available ELISA kits (88-7013; Invitrogen, Thermo Fisher Scientific, United States) according to the manufacturer’s instructions. The sensitivity of the ELISA kits was 8 pg/ml and the standard cure range was 8–1,000 pg/ml.

### Western Blot

The Western blotting was performed as previously described (Wei et al., 2018). Briefly, the hippocampal samples were homogenized on ice in 1 ml of ice-cold RIPA buffer containing the protease inhibitor phenylmethanesulfonyl fluoride (2 mM). The lysates were centrifuged at 13,000 rpm at 4°C for 30 min to eliminate cellular debris. The protein concentrations in the supernatants were determined using a BCA protein assay kit. Equal amounts of proteins (40 μg) per lane from each sample were separated using 12% SDS PAGE gels and then transferred onto polyvinylidene fluoride membranes (Millipore, Boston, MA, United States). The membranes were blocked with 5% skimmed milk in Tris-buffered saline with Tween detergent for 1 h and incubated at 4°C overnight with primary antibodies, including rabbit anti-BDNF antibody (dilution 1:1000, GB11559, Servicebio, Wuhan, China) and mouse anti-β-actin antibody (dilution 1:3000, GB12001, Servicebio, Wuhan, China). After three 5 min washes, the membranes were incubated with an HRP-conjugated secondary antibody for 1 h, followed by incubation with enhanced chemiluminescence Western blot substrate (Millipore, Billerica, MA, United States). Protein bands were detected and analyzed using C-Digit software (LI-COR, United States). The relative expression of BDNF was normalized to that of β-actin.

### Morris Water Maze (MWM)

Hippocampus-dependent spatial learning and memory were evaluated by the MWM test including 5 days-spatial acquisition trials and 1 day-probe trials, as described in our previous studies (Wei et al., 2015). In the spatial acquisition trials, the mice needed to learn how to find the hidden platform by using distinctive distal visual cues around the tank. Each animal was placed in a fixed starting position in the pool facing the tank wall. They were allowed to locate the platform for 60 s until the target was reached. Otherwise, beyond the time limit, mice were guided to the platform and they remained in that position for 30 s. Four place trials per day in different quadrants were conducted and the platform remained unchanged during the acquisition phase. Parameters including escape latency (time to reach the platform) and swimming speeds were recorded during the spatial acquisition trials. In the probe trials, the platform was removed from the first quadrant, and the reverse quadrant was used as the starting position to evaluate the reference memory. The mice were allowed to swim freely for 60 s. The probe parameters including platform crossings and time, were recorded in the target quadrant.

### Statistical Analysis

Data were analyzed using Statistical Program for Social Sciences software (version 20.0; SPSS Inc., Chicago, IL, United States). Graphs were drawn using GraphPad Prism version 5.0 statistical package (GraphPad Software Inc.). Data are presented as the mean ± standard deviation (SD). The escape latency and swimming speeds in the spatial acquisition trials were analyzed using a two-way repeated-measures analysis of variance (ANOVA). Parameters of the probe trials and data from ELISA, Western blotting, and immunofluorescence were analyzed using one-way ANOVA followed by *post hoc* Tukey multiple comparisons. Significant difference was set at *p* < 0.05.

## Results

### Surgery and Anesthesia Significantly Inhibit α7nAChR Expression in the Hippocampus and Cortex in Aged Mice

**FIGURE 2:**
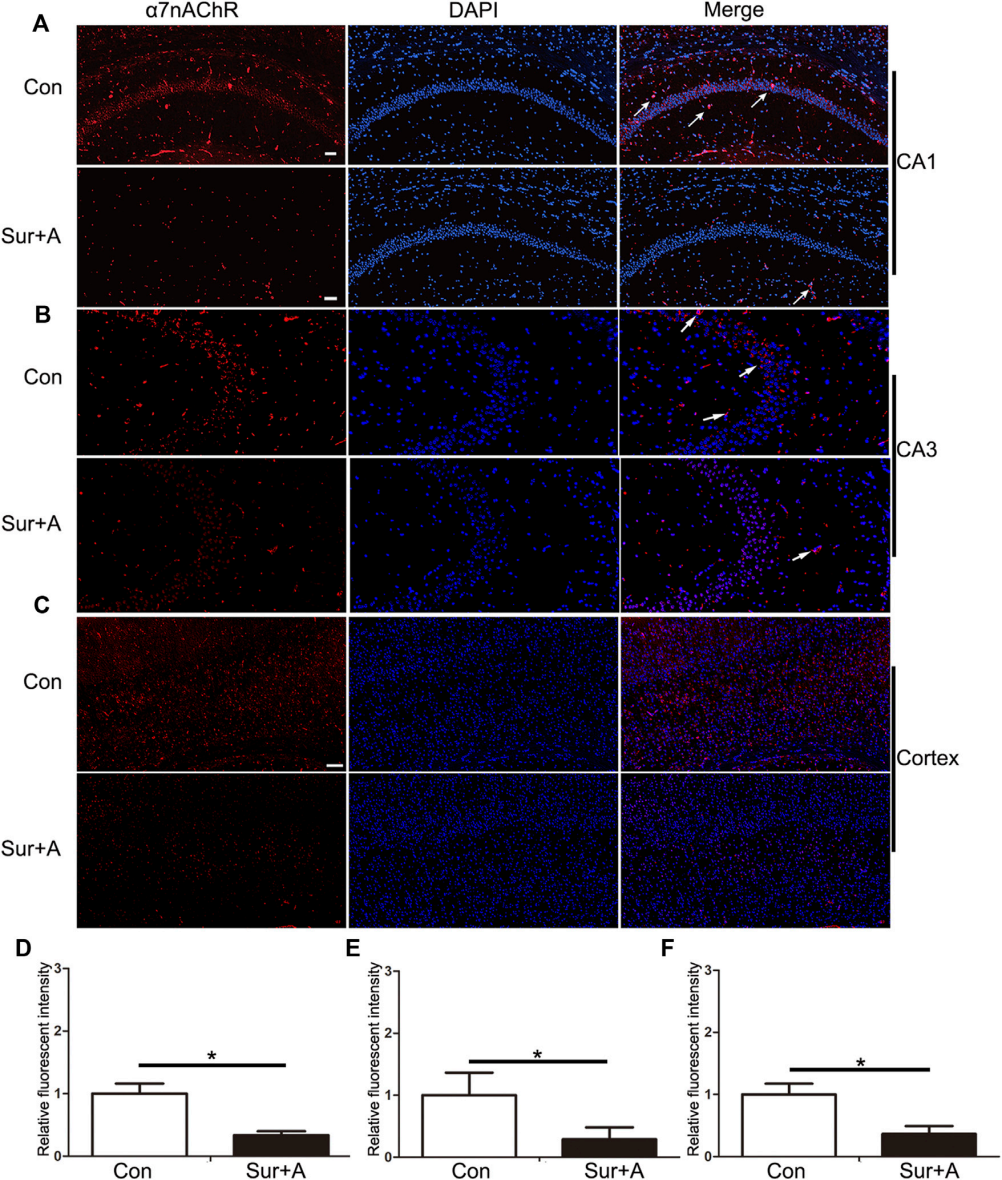
Effects of surgery and anesthesia on α7nAChR expression in the hippocampus and cortex in aged mice. Representative images of α7nAChR positive cells (red) in the hippocampi of CA1 region **(A)**, CA3 region **(B)**, and cortex **(C)**, 24 h after surgery and anesthesia. Quantifications of the relative fluorescent intensity for α7nAChR positive cells in hippocampal CA1 region **(D)**, and CA3 region **(E)**, and cortex **(F)**. Data are presented as mean ± SD. DAPI staining is shown in blue. *p < 0.05. Scale bar for CA 1 = 50 μm; Scale bar for cortex = 100 μm. Con = Control; Sur+A, Surgery plus Anesthesia.

Previous studies have shown that α7nAChR stimulation in peripheral macrophages prevents NF-κB activation thereby preventing cognitive impairment induced by surgery and anesthesia (Terrando et al., 2011). However, whether surgery and anesthesia affect α7nAChR expression in the CNS remains unclear. We assessed the expression levels of α7nAChR in the hippocampus and cortex of aged mice with PNDs by immunofluorescence, 24 h after surgery. The results revealed that surgery and anesthesia significantly decreased α7nAChR expression in the hippocampal Cornu Ammonis 1 (CA1) region (0.34 ± 0.06 vs 1.00 ± 0.16, *p* < 0.0001; *n* = 6/group, Figures 2A,D), hippocampal CA 3 region (0.29 ± 0.19 vs 1.00 ± 0.36, *p* = 0.02; *n* = 6/group, Figures 2B,E), and cortex (0.36 ± 0.12 vs 1.00 ± 0.17, *p* < 0.0001; *n* = 6/group; Figures 2C,F).

### α7nAChR Stimulation Mediates Systemic and Hippocampal Anti-inflammatory Effects

**FIGURE 3:**
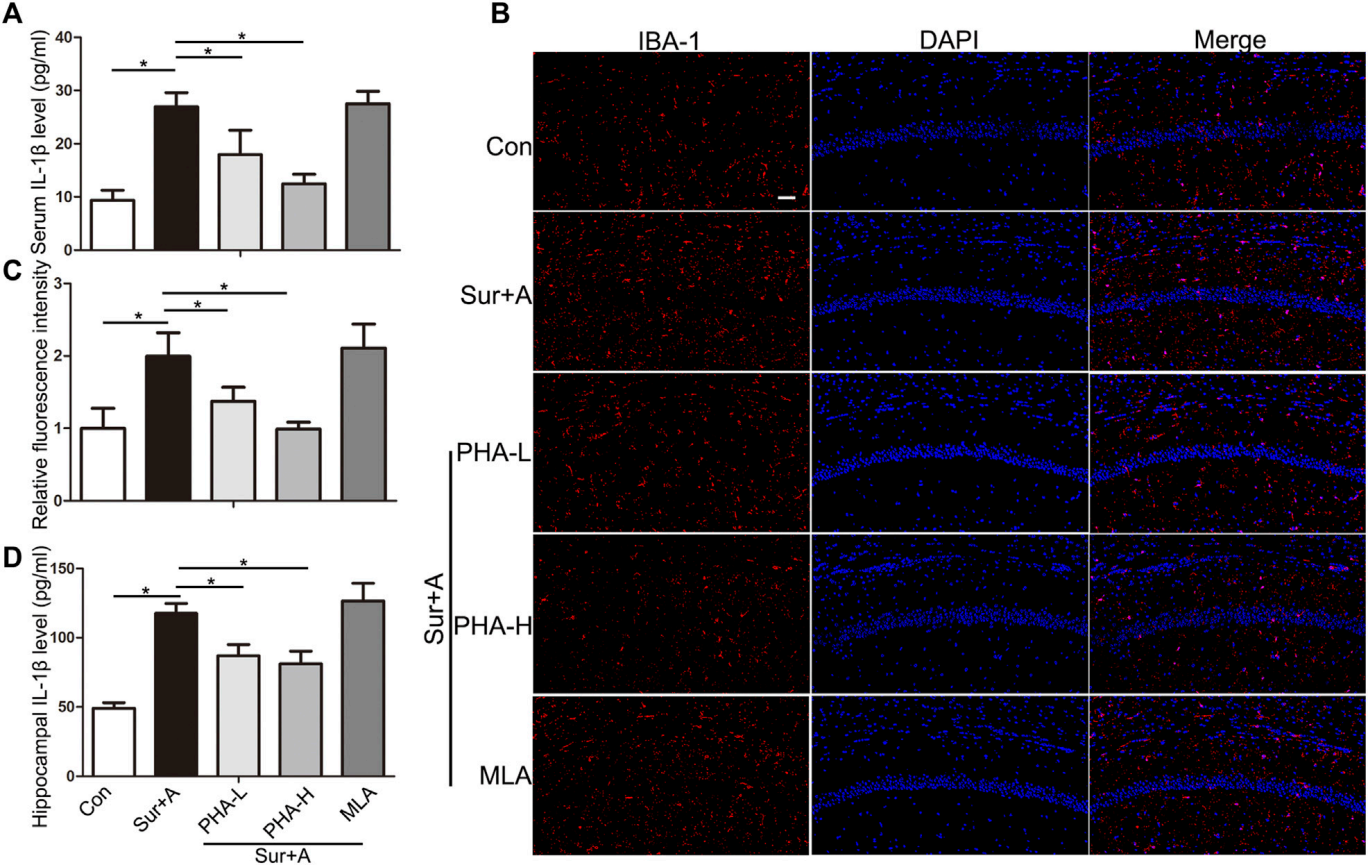
Effects of α7nAChR stimulation on systemic and hippocampal inflammatory response in aged mice. **(A)** Levels of IL1β in the serum 24 h after surgery. **(B)** Representative images of IBA-1 positive microglia (red) in the hippocampi of CA1 region. **(C)** Quantifications of the relative fluorescent intensity for IBA-1 positive microglia in hippocampal CA1 region. **(D)** Levels of IL1β in the hippocampus 24 h after surgery. Data are presented as mean ± SD. DAPI staining is shown in blue. *p < 0.05. Scale bar = 50 μm. Con, Control; Sur+A, Surgery plus Anesthesia; PHA-L, low-dose PHA (0.3 mg/kg); PHA-H, high dose PHA (0.6 mg/kg).

Hippocampus, one of brain regions mostly susceptible to surgical and anesthesia stress, are central players in cognitive processes of PNDs (Wei et al., 2019; Yang et al., 2020). Therefore, we focused on the hippocampus in subsequent studies. We tested the effects of PHA, a selective α7nAChR agonist, on microglial activation and IL-1β expression in serum and hippocampus. Following surgery and anesthesia, IL-1β in serum was significantly increased (26.96 ± 2.64 pg/ml vs 9.39 ± 1.84 pg/ml, *p* < 0.0001; *n* = 4/group, Figure 3A). Pretreatment with two doses of PHA (PHA-L: low-dose 0.3 mg/kg, PHA-H: high-dose 0.6 mg/kg), reduced the serum level of IL-1β (PHA-L: 17.95 ± 4.57 pg/ml vs 26.96 ± 2.64 pg/ml, *p* = 0.003; PHA-H: 12.49 ± 1.76 pg/ml vs 26.96 ± 2.64 pg/ml, *p* < 0.0001, *n* = 4/group, Figure 3A). Twenty-four hours after surgery, microglial activation markedly increased in hippocampus, as evidenced by the significant upregulation of IBA-1 in CA1 region (1.99 ± 0.32 vs 1.00 ± 0.26, *p* = 0.001; *n* = 4/group, Figures 3B,C). Administration of PHA inhibited the surgery and anesthesia-induced activation of macroglia (PHA-L: 1.37 ± 0.19 vs 1.99 ± 0.32, *p* = 0.028, PHA-H: 0.99 ± 0.09 vs 1.99 ± 0.32, *p* = 0.001; *n* = 4/group, Figures 3B,C). Pretreatment with methyllycaconitine (MLA), a selective α7nAChR antagonist, did not significantly promote hippocampal microglial activation, 24 h after surgery (2.11 ± 0.33 vs 1.99 ± 0.32, *p* = 0.96; *n* = 4/group, Figures 3B,C). Consistent with these findings, hippocampal IL-1β expression assessed by ELISA was also enhanced 24 h after surgery (117.60 ± 7.22 pg/ml vs 49.09 ± 3.95 pg/ml, *p* < 0.0001; *n* = 4/group, Figure 3D), which was reversed by pretreatment with PHA pretreatment (PHA-L: 86.93 ± 8.17 vs 117.60 ± 7.22 pg/ml, *p* = 0.001, PHA-H: 81.21 ± 9.12 vs 117.60 ± 7.22 pg/ml, *p* < 0.0001; *n* = 4/group, Figure 3D).

### α7nAChR Regulated BDNF Expression in the Hippocampus

**FIGURE 4:**
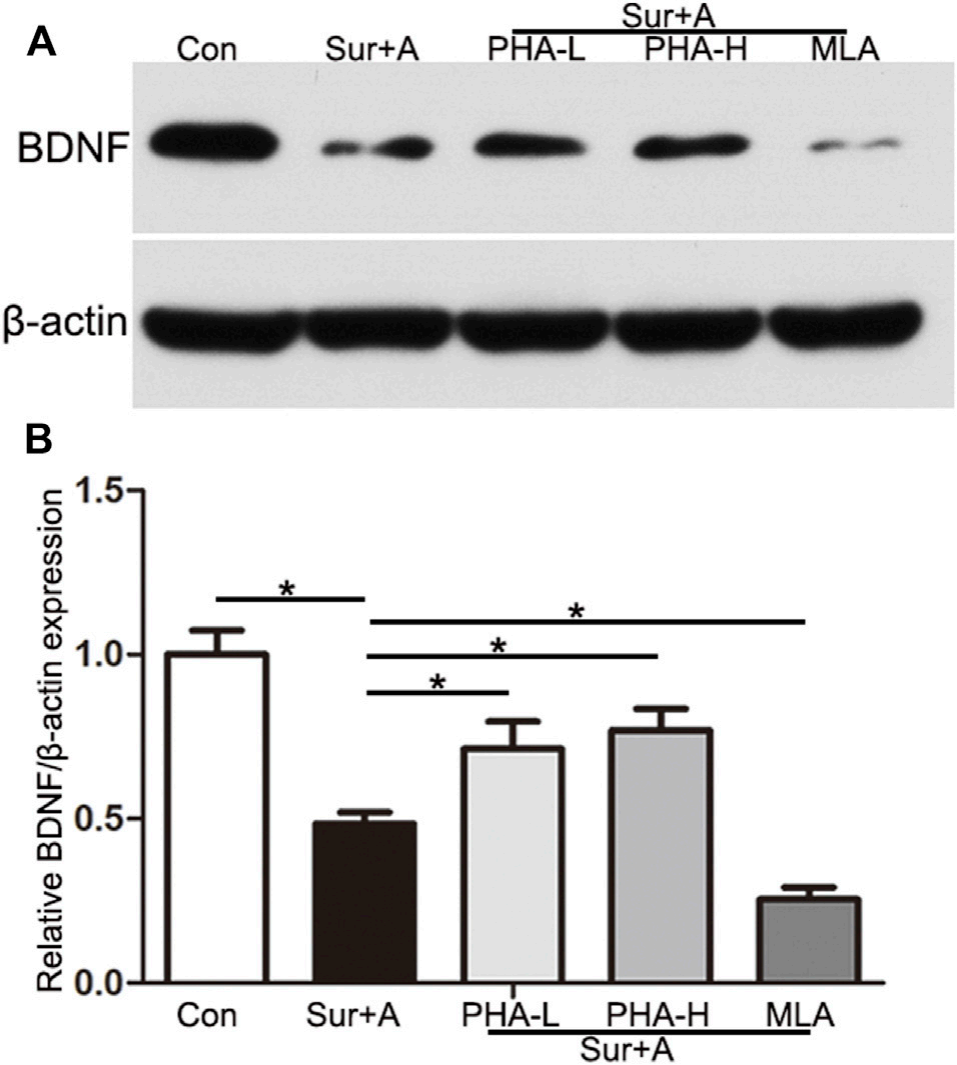
α7nAChR mediates BDNF expression in the hippocampus. **(A)** Representative Western blots of brain-derived neurotrophic factor (BDNF) in the hippocampus 2 h after surgery. β-actin was used as a loading control. **(B)** Quantitative analysis of BDNF levels. Data are presented as the mean ± SD. *p < 0.05. Con, Control; Sur+A, Surgery plus Anesthesia; PHA-L, low-dose PHA (0.3 mg/kg); PHA-H, high-dose PHA (0.6 mg/kg).

To explore the mechanism by which α7nAChR in the CNS mediates neuroprotection in PNDs, we administered PHA and MLA, agonist and antagonist of α7nAChR, and evaluated BDNF levels in the hippocampus by immunoblotting. Compared with those in the control group, the levels of hippocampal BDNF in the surgery and anesthesia group were significantly decreased (0.48 ± 0.03 vs 1.00 ± 0.07, *p* < 0.0001; *n* = 4/group, Figure 4). Single-dose administration of PHA reversed downregulation induced by the surgery and anesthesia (PHA-L: 0.71 ± 0.08 vs 0.48 ± 0.03, *p* = 0.001, PHA-H: 0.77 ± 0.07 vs 0.48 ± 0.03, *p* < 0.0001; *n* = 4/group, Figure 4). Conversely, preoperative administration of a single-bolus dose of the selective an antagonist of α7nAChR (MLA) further inhibited BDNF expression in the hippocampus after surgery and anesthesia, suggesting a key role for α7nAChR in mediating BDNF levels in PNDs (0.25 ± 0.04 vs 0.48 ± 0.03, *p* = 0.001; *n* = 4/group, Figure 4).

### α7nAChR Stimulation Prevents PNDs Through BDNF-dependent Mechanism in Aged Mice

**FIGURE 5:**
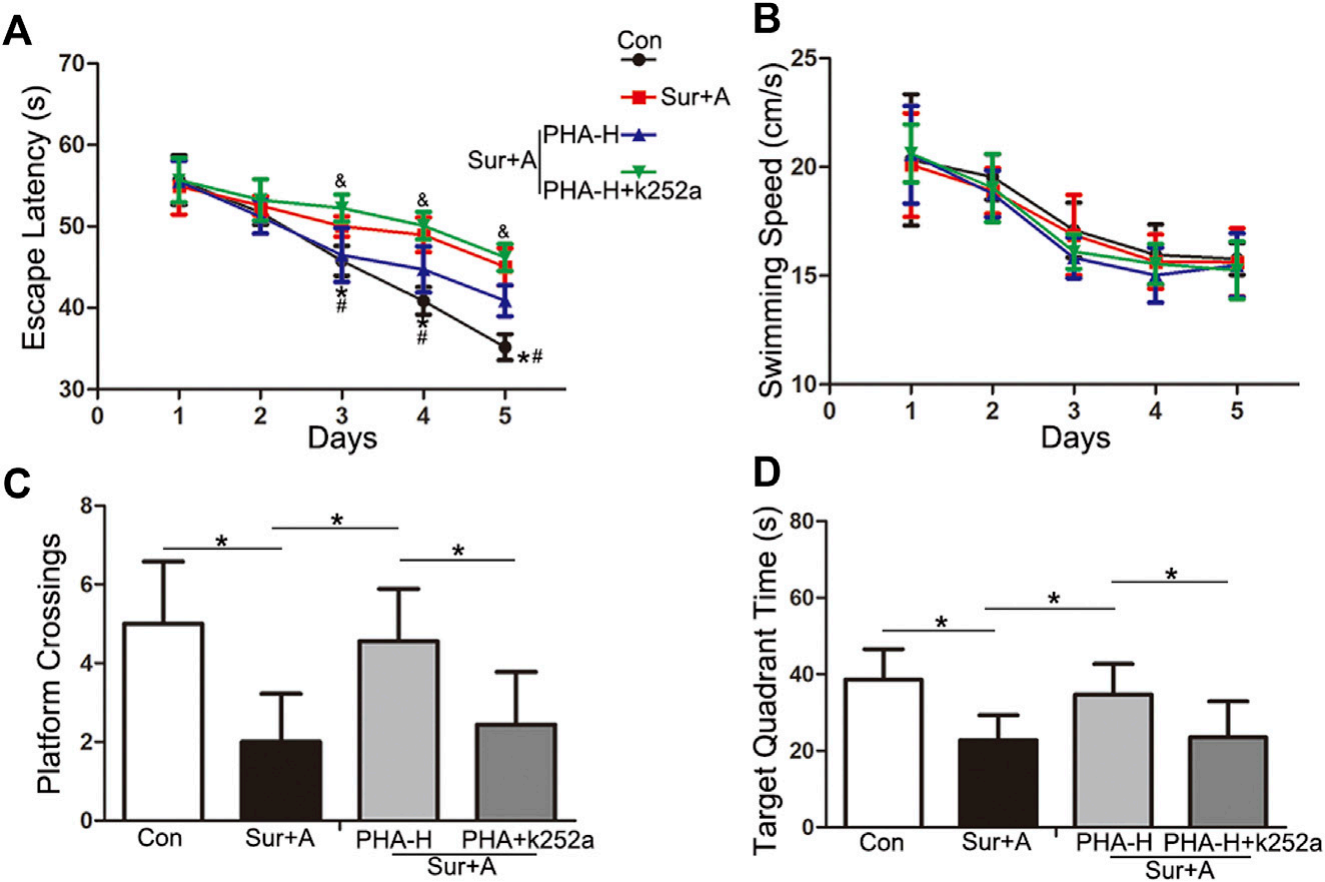
Effects of α7nAChR modulation on hippocampal-dependent spatial learning and memory, following surgery and anesthesia in aged mice. The MWM test consists of 5 days-spatial acquisition trials **(A**,**B)** and 1 day-probe trials **(C**,**D)**. Data are presented as mean ± SD. **(A)** Escape latency to reach the platform. *p < 0.05 versus the control groups, ^#^ *p* < 0.05 versus the surgery plus anesthesia group, ^&^ *p* < 0.05 versus the K-252a group. **(B)** Average swimming speed of each group during the acquisition phase. **(C)** The number of platform crossings with a 60-s limit for each group in the probe trials. **(D)** Time spent in the target quadrant for each group with a 60-s limit for each group in the probe trials. *p < 0.05 Con, Control; Sur+A, Surgery plus Anesthesia; PHA-H, high-dose PHA (0.6 mg/kg).

Next, we explored the effects of the α7nAChR/BDNF signaling pathways on hippocampal-dependent memory function after surgery using the MWM test. Animals were administered a selective antagonist of the BDNF receptor TrkB (K-252a), before surgery. Comparisons using two-way repeated-measures ANOVA were performed for the escape latency and swimming speeds. Significant difference in the escape latency was observed between the control and anesthesia plus surgery groups at days 3, 4, and 5 during the spatial acquisition trials (days 3: 50.00 ± 1.25 s vs 45.78 ± 1.83 s, *p* = 0.001; days 4: 48.96 ± 2.12 s vs 40.86 ± 1.66 s, *p* < 0.0001; days 5: 45.04 ± 2.28 s vs 35.20 ± 1.60 s, *p* < 0.0001; *n* = 9/group, Figure 5A). α7nAChR stimulation significantly improved cognitive performance compared with anesthesia plus surgery group during the spatial acquisition (days 3: 46.50 ± 3.32 s vs 50.00 ± 1.25 s, *p* = 0.001; days 4: 44.72 ± 2.85 s vs 48.96 ± 2.12 s, *p* = 0.032; days 5: 40.88 ± 1.90 s vs 45.04 ± 2.28 s, *p* = 0.02; *n* = 9/group, Figure 5A). However, pretreatment with K-252a abolished the α7nAChR-induced neuroprotection (days 3: 0.25 ± 0.04 vs 46.50 ± 3.32 s, *p* = 0.018; days 4: 0.25 ± 0.04 vs 44.72 ± 2.85 s, *p* = 0.031; days 5: 0.25 ± 0.04 vs 40.88 ± 1.90 s, *p* = 0.004; *n* = 9/group, Figure 5A). Comparisons among the four groups revealed no significant differences in swimming speeds, to exclude possible locomotor impairments (*p* > 0.05, Figure 5B).

In the following probe trials, platform crossings and target quadrant times were analyzed using a one-way ANOVA. Compared with that in the control group, significant decrease in platform crossings (2.00 ± 1.23 vs 5.00 ± 1.58, *p* < 0.0001; *n* = 9/group, Figure 5C) and target quadrant time (22.80 ± 6.50 s vs 38.56 ± 8.01 s, *p* = 0.001; *n* = 9/group, Figure 5D) were observed in surgery plus anesthesia group on day 12 following surgery. PHA pretreatment attenuated surgery-induced decrease in platform crossings (4.56 ± 1.33 vs 2.00 ± 1.23, *p* = 0.002; *n* = 9/group, Figure 5C) and target quadrant time (34.72 ± 7.96 s vs 22.80 ± 6.50 s, *p* = 0.018; *n* = 9/group, Figure 5C). K-252a administration abolished the above attenuation (platform crossings: 2.44 ± 1.33 vs 4.56 ± 1.33, *p* = 0.013; target quadrant time: 23.51 ± 9.37 s vs 34.72 ± 7.96 s, *p* = 0.028; *n* = 9/group, Figure 5D).

## Discussion

**FIGURE 6:**
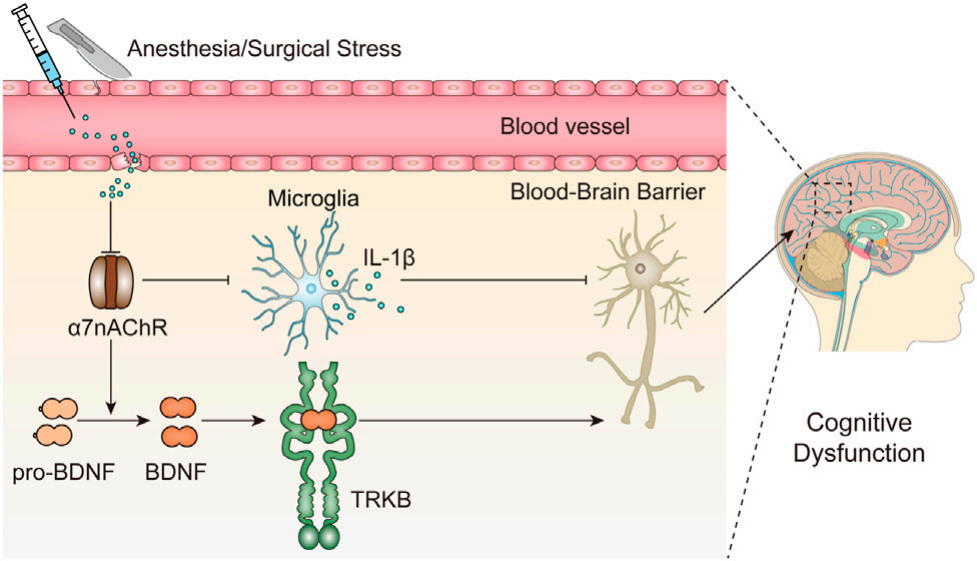
Working hypothesis of the role α7nAChR-mediated neuroprotection in PNDs. Surgery and anesthesia lead to a wide range of inflammatory cytokines in the serum, such as IL-1β, and disrupts the blood-brain barrier integrity, which may facilitate the downregulation of α7nAChR in the brain, in particular, but not restricted to the hippocampus. Subsequently, the inhibition of α7nAChR triggers a cascade of downstream signaling events including proinflammatory responses (IL-1β) and BDNF decrease and may further cause neuronal apoptosis, consequently contributing to cognitive dysfunction.

The results presented here indicate that surgical trauma and anesthesia upregulate peripheral IL-1β expression and inhibit α7nAChR expression in the hippocampus and cortex. α7nAChR downregulation promotes microglial activation and subsequent IL-1β upregulation, suppresses BDNF expression in the hippocampus and may further cause neuronal apoptosis, consequently contributing to cognitive dysfunction in aged mice (Figure 6).

### Neuroprotective Roles of α7nAChR-Mediated Cholinergic Pathway in PNDs

Cholinergic transmission mediated by nAChRs has a significant effect on cognitive function. Degeneration of the cholinergic pathway, combined with aberrant nAChR expression, results in age-related cognitive impairment (Hoskin et al., 2019). The modulation of nAChRs, particularly α7nAChR, has been demonstrated to treat nervous-system disorders, including schizophrenia as well as depression, and neurodegenerative diseases including AD and Parkinson’s disease (Taly et al., 2009; Hoskin et al., 2019). α7nAChR, one of the most predominant subtypes found in the mammalian brain, is formed through homomeric α-type subunit configurations and is the most commonly targeted receptor subtype for cognitive dysfunction to date (Taly et al., 2009; Terry and Callahan, 2019). It is widely localized in the glia, glutamatergic neurons, and GABAergic interneurons in the hippocampus and prefrontal cortex, and its activation can potentiate hippocampal-prefrontal cortex synapses (Stoiljkovic et al., 2016). A massive reduction in α7nAChR is reportedly associated with progressive cognitive decline in AD (Bertrand and Terry, 2018). However, to date, no studies have shown the effects of surgery and anesthesia on α7nAChR expression in the brain. Our results revealed that surgery and anesthesia suppressed the expression of α7nAChR in the hippocampus and cortex, as evidenced by the significant reduction in α7nAChR-positive cells in the CA1, CA3, and cortex (Figure 2), which may be a key factor for uncontrolled neuroinflammation and subsequent cognitive impairment. Although the mechanism by which upstream putatively inhibits the expression of α7nAChR was beyond the scope of this study, it is notable that Aβ-α7nAChR interaction was observed in rodent experimental models that may lead to an atomic structure damage and significant reduction in α7nAChR in AD, as evidenced by Aβ enrichment in α7nAChR-abundant regions (Wang et al., 2000; Farhat and Ahmed, 2017; Hoskin et al., 2019). Aβ accumulation in the CNS, induced by surgery and anesthesia, is also a hallmark of PNDs (Xie et al., 2013; Zhang et al., 2020). Neuroinflammation and reactive oxygen species resulting from the surgery and anesthesia have been hypothesized to inhibit α7nAChR expression; however, this remains to be experimentally explored (Wei et al., 2019).

### Anti-inflammatory Response Induced by α7nAChR Stimulation in the Periphery and Hippocampus

Surgery and anesthetics spark systemic inflammatory cytokines and damage the blood-brain barrier, thereby sending harmful chemicals and cells into the brain and activating microglia, which further contribute to neuroinflammation (Mahanna-Gabrielli et al., 2019; Yang et al., 2020). The specificity of reactive microgliosis and IL-1β involvement in PNDs has been revealed by previous studies (Luo et al., 2021). Circulating IL-1β and hippocampal IL-1β levels are consistently upregulated after surgery and anesthesia, in preclinical models, and in surgical patients (Skvarc et al., 2018). IL-1β neutralizing antibodies or knockout of the IL-1 receptor can improve postoperative cognitive impairment in aged mice, suggesting the necessity and importance of producing PNDs (Cibelli et al., 2010; Barrientos et al., 2012). Maze et al. highlighted the importance of peripheral α7nAChR in resolving cognitive decline following tibial fracture surgery under general anesthesia. Activation of α7nAChR in macrophages prevents TNFα-induced NF-κB activation and macrophage migration into the hippocampus (Terrando et al., 2011). We have previously reported that nicotine, a non-selective nAChRs agonist, suppresses IL-1β levels in the circulation and hippocampus, and attenuates the surgery-induced cognitive dysfunction (Wei et al., 2018). Herein, we reported that a specific agonist of α7nAChR (PHA) reduces systemic and hippocampal IL-1β expression, and microglial activation in response to surgery and anesthesia in two intraperitoneal doses (0.3 and 0.6 mg/kg) (Figure 3). The nod-like receptor pyrin domain-containing 3 inflammasome may be a pivotal target for α7nAChR stimulation-mediated IL-1β downregulation (Wei et al., 2019). Although it was anticipated that the α7nAChR blocker would worsen the IL-1β mediated inflammatory response to surgery and anesthesia, the results of this study revealed no significant difference between the surgery plus anesthesia and MLA treatment groups (Figure 3). A possible explanation is that the hippocampal infiltration of peripheral macrophages derived from myeloid cells is much less in our laparotomy aged mice model than that in the tibial fracture adult mice model (Terrando et al., 2011). In their study, Maze et al. found that MLA pretreatment at the same dose could promote large number of monocyte-derived macrophages into the disturbed blood-brain barrier and induce neuroinflammation after surgery (Terrando et al., 2011). Another possibility is that the MLA dosage was low in our laparotomy PNDs model, and the α7nAChR blocker induced a feed-forward response to overcome the blockade.

### Regulation of α7nAChR on BDNF Expression in the Hippocampus

Neurotrophins, a family of growth factors including NGF, BDNF, NT-3, and NT-4 are responsible for the regulation of neuronal progression, differentiation, and durability in the peripheral and CNSs. These proteins trigger and regulate neurogenesis, which is the ability of existing NSCs to generate new nerve cells (Girotra et al., 2022). BDNF protein synthesized in the cell bodies of neurons and glia, in which the α7nAChR are expressed, is widely distributed throughout the brain but is expressed in the highest amounts in the hippocampus, primarily by excitatory neurons including the pyramidal neurons in the CA1-CA3 regions and dentate granule cells (Zaletel et al., 2017; Wang et al., 2022). BDNF can activate the TrkB receptor and trigger downstream signaling pathways that modulate neuronal function and synaptic processes (Wang et al., 2022). BDNF has emerged as a critical factor underlying the pathogenesis of PNDs. We and other researchers have reported that dysregulation of BDNF/TrkB signaling leads to cognitive impairment after surgery and anesthesia, and BDNF upregulation by neuroprotective agents rescued this deficit (Wei et al., 2018; Qiu et al., 2020; Yin et al., 2022). However, the signaling pathways upstream of BDNF remain unclear in PNDs. In this study, we found that downregulated BDNF levels were evident 24 h after surgery, and BDNF expression in the hippocampus was specifically modulated by PHA and MLA, suggesting a potential role of α7nAChR in BDNF expression (Figure 4). Using the MWM test, we further observed that the BDNF/TrkB signaling blocker abolished cognitive improvement induced by the α7nAChR agonist (Figure 5). The current results are consistent with previous reports on AD. A recent study found that co-treatment with MLA promoted the reduction of BDNF in the hippocampus and worsened Aβ-induced cognitive impairment, suggesting a determinant role of α7nAChR in BDNF expression and memory recovery in AD (Telles-Longui et al., 2019). Chen et al. has also reported that α7nAChR blockade could inhibit the reverse of hippocampal BDNF and abolish the neuroprotective effects of simvastatin in Aβ_25-35_-mice, an inhibitor of 3-hydroxy-3-methyl-glytarylcoenzyme reductase, which is effective in improving cognition in AD patients (Wang et al., 2015).

### α7nAChR Being Developed as a Potential Therapeutic Target for Age-Related Neurodegenerative Diseases

nAChRs are widely dispersed throughout the CNS, making them a reasonable target for treating nervous system disorders, including neurodegenerative diseases. Several clinical trials have been conducted over the past two decades. A pilot trial found that pozanicline (ABT-089), a partial agonist that binds with high affinity to α4β2 nAChR, was well tolerated in human subjects and was shown to be effective in treating adult attention-deficit/hyperactivity disorder (Wilens et al., 2006). Encenicline, a partial selective agonist of the α7nAChR having a long half-life, has been reported to improve neuropsychological test performance in AD patients (Hoskin et al., 2019). Furthermore, galantamine, an anticholinesterase inhibitor, has modulatory effects on the α7nAChR and was also useful for schizophrenia patients in a case study (Rosse and Deutsch, 2002). However, potentiating adverse effects, particularly a major gastrointestinal issue in elderly patients, hampered the development of nAChR agonists as a potential treatment option in many clinical trials. More specific new biologics that target the α4β2 or α7nAChR, which may have fewer non-CNS adverse effects, remain to be developed and investigated (Hoskin et al., 2019). Future potential application of nAChRs, particularly α7 subunit agonist application, during the perioperative period to prevent the PNDs, needs to be explored and awaits further preclinical and clinical evidence.

## Conclusion

The data collected in this study demonstrated that α7nAChR mediated neuroprotective effects in PNDs of aged mice, and the underlying mechanism might be associated with suppression of the IL-1β inflammatory pathway and activation of the BDNF/TrkB signaling pathway in the hippocampus. These data reinforce future studies concerning α7nAChR as a pharmacological target to prevent PNDs; however, clinical application awaits further definitive evidence and safety testing.

## Data Availability

The original contributions presented in the study are included in the article/Supplementary Material, further inquiries can be directed to the corresponding author.
